# Selection of Dental Implants and Restorations Among Dentists in the Kingdom of Saudi Arabia: A Cross-Sectional, Questionnaire-Based Survey

**DOI:** 10.7759/cureus.57242

**Published:** 2024-03-30

**Authors:** May W Al-Khudhairy, Afnan M Alharbi, Mahesh Suganna, Hina Kausher, Shaden Alshammari, Nouf Alsaleh, Fatemah Allmaie

**Affiliations:** 1 Department of Oral and Maxillofacial Surgery and Diagnostic Sciences, College of Medicine and Dentistry, Riyadh Elm University, Riyadh, SAU; 2 Department of Dentistry, College of Medicine and Dentistry, Riyadh Elm University, Riyadh, SAU; 3 Department of Prosthodontics, College of Medicine and Dentistry, Riyadh Elm University, Riyadh, SAU; 4 Department of Prosthodontics, Riyadh Elm University, Riyadh, SAU

**Keywords:** implant planning software, cross-sectional survey, dentists, implant systems, loading protocols, restoration, dental implants

## Abstract

Background

Dental implants play a crucial role in modern dentistry, offering a durable and aesthetic option for tooth replacement. Understanding the preferences and practices of dentists regarding implant selection and restoration can provide critical insights into current trends and potential areas for improvement. As such, the objective of this study was to discover characteristics determining the selection criteria and preferences for dental implants and restorations among Saudi Arabian dentists.

Methodology

A cross-sectional, questionnaire-based survey was conducted among Saudi Arabian dentists to assess their practices and preferences for dental implant selection and restoration. The survey explored variables, including age, gender, educational status, regional practice distribution, implant-related experience, loading protocols, and implant system preferences.

Results

A total of 742 dental professionals responded to our questionnaire. The study revealed that a significant majority of Saudi Arabian dentists had placed (78.7%) and restored (72.9%) implants. Most dentists (78.6%) had participated in implant treatment planning. However, consistent usage of implant planning software was relatively low (29.8%). Loading protocol preferences varied, with early loading favored for anterior teeth and immediate loading for posterior teeth and edentulous patients. The main deterrents to immediate loading were patient type (27.0%), lack of training (19.9%), additional surgeries (19.9%), and administrative restrictions (17.8%). Straumann was the most preferred implant system, with aesthetic outcomes considered the most important factor in system selection.

Conclusions

The study provides a comprehensive overview of dental implant practices among Saudi Arabian dentists. It highlights a strong emphasis on aesthetic outcomes, a diverse approach to loading protocols, and room for increased usage of implant planning software. The findings suggest potential areas for further training and support, particularly in the use of immediate loading and implant planning software.

## Introduction

During the dawn of the 1980s, a remarkable breakthrough occurred in the dental health sector with the advent of osseointegrated dental implants [1]. These became increasingly popular in the United States, demonstrating a significant rise in adoption over the years [2]. A retrospective cohort study was conducted to investigate the success rates of these implants when used in conjunction with crowns and fixed partial prostheses. The outcomes indicated a variance in success rates, with lower results observed among general dental practitioners in private practices as opposed to specialists in university or specialty settings. A similar pattern was noted in a Swedish study, where peri-implantitis seemed more prevalent when the treatment was performed by general dental practitioners [3]. As a reliable alternative to conventional dentures, dental implants offer numerous benefits, including improved stability, enhanced masticatory function, and significant improvements in quality of life [4-6]. The selection of dental implants and their restorations, however, can vary widely among practitioners due to differences in training, experience, and personal preference [6-9].

In the Kingdom of Saudi Arabia (KSA), dental implants are increasingly recognized as a preferred treatment option for tooth loss [10]. The adoption and success rates of these procedures have grown substantially, reflecting advancements in dental health practices and increased patient awareness [11,12]. Despite this growth, comprehensive data regarding the selection and application of dental implants and restorations by dental practitioners in the KSA remains limited. Understanding the preferences and practices of dental practitioners in the KSA regarding dental implants and their restorations is key to identifying potential gaps in knowledge, improving dental education, and optimizing patient outcomes. Keeping all the above factors in mind, the purpose of this study was to identify determinants impacting the selection criteria and preferences for dental implants and restorations among Saudi Arabian dentists.

## Materials and methods

Study design and setting

The study adopted a cross-sectional design with a focus on dental practitioners in Saudi Arabia. The geographical focus of the investigation was the KSA. The subjects of interest were dental practitioners in Saudi Arabia, and the recruitment process involved a probabilistic sampling approach to ensure random selection. The sample universe encompassed certified dentists from both government and private dental colleges, as well as practitioners registered with the Saudi Commission for Health Specialties. The primary research question was: “Did any factors influence the selection criteria and preferences for dental implants and restorations among dentists in Saudi Arabia?”

Study hypotheses

The null hypothesis for this study was that no identifiable factors influenced the selection criteria and preferences for dental implants and restorations among dentists in Saudi Arabia. Alternatively, it was hypothesized that certain factors did influence the selection criteria and preferences for dental implants and restorations among dentists in Saudi Arabia.

Ethical approval

The research proposal was subjected to review by the Institutional Review Board (IRB) at Riyadh Elm University (REU) and strictly adhered to the IRB’s guidelines and protocols. The IRB’s approval was granted in September 2023 (approval number: FIRP/2023/133/1027/924).

Data collection protocol

The data collection instrument for this study was a questionnaire, which had been refined based on a previous study [1] and expert opinions. A group of 25 individuals, representative of the study population, participated in a pilot test to verify the questionnaire’s clarity and reliability. The questionnaire was also subjected to validity tests by the Department of Prosthodontics.

Data collection tools

The questionnaire encompassed demographic information (age, gender, and educational status) and 16 structured questions regarding implant dentistry practices. The questionnaire sections included implant training and experience, implant treatment planning, implant restorations, implant system preference and selection, and implant loading protocol. The selection of different provinces across Saudi Arabia aimed to ensure geographical representation. This approach was designed for the generalization of the study’s findings, not for inter-province comparison.

Outcomes assessed

The outcome variables were the preferences and trends in implant dentistry practices among practitioners in Saudi Arabia. This included the types of implants and restorations used, selection criteria, loading protocols, and the usage of implant planning software.

Sample size determination

A convenience sampling approach was employed. The required sample size was estimated at a minimum of 380 participants, calculated with a 5% margin of error, a 95% confidence level, a dentist population of 27,181 [13], and a presumptive response distribution of 50%. This calculation was determined using the Raosoft online sample size calculator.

Eligibility criteria

Eligible participants were postgraduate dentists and practitioners affiliated with various dental colleges across Saudi Arabia. Participation was voluntary, with assurances of confidentiality. The questionnaire was distributed electronically via a Google Docs link. BDS students and interns who were not actively involved in implant treatment procedures or those who declined participation upon contact were excluded.

Statistical analysis

The data were processed and analyzed using SPSS Statistics for Windows, version 25.0 (IBM Corp., Armonk, NY, USA). Descriptive analysis was pursued, followed by inferential statistical methods. Categorical data was compared using chi-square and Fisher’s exact tests. A p-value ≤0.05 at a 95% confidence interval was considered statistically significant.

## Results

The sample size for this study encompassed a total of 742 dental professionals. Delving into the demographic parameters (Table 1), the study revealed a diverse distribution across various age brackets, indicative of the heterogeneous nature of the participant pool. The largest segment was 271 (36.5%) individuals aged 24-30 years, constituting 36.5% of the sample, followed by those aged 31-35 years at 187 (25.2%) respondents, and 36-40 years at 121 (16.3%) respondents. Regarding gender representation, the cohort demonstrated a balanced split, with 355 (47.8%) male and 387 (52.2%) female participants. However, the educational background of participants varied, with postgraduates comprising 354 (47.7%) individuals of the sample, 227 (30.6%) graduates, and the rest holding a PhD, indicating a diverse range of expertise levels. Geographically, participants were distributed across different regions of Saudi Arabia. The southern region had the highest representation at 181 (24.4%) individuals, followed by the eastern region at 146 (19.7%), the northern region at 141 (19.0%), the western region at 18.6%, and the rest at 136 (18.3%) respondents from the central region.

**Table 1:** Demographic parameters assessed.

Variable type analyzed		Frequency n (%)	Chi-square value
Age ranges analyzed (in years)	24–30 years	271 (36.5%)	23.57
	31–35 years	187 (25.2%)	
	36–40 years	121 (16.3%)	
	41–50 years	117 (15.8%)	
	51–55 years	46 (6.2%)	
Gender	Male	355 (47.8%)	18.92
	Female	387 (52.2%)	
Dental educational status	Graduate	227 (30.6%)	12.76
	PhD	161 (21.7%)	
	Postgraduate	354 (47.7%)	
Region of practice in Saudi Arabia	Northern part	141 (19.0%)	17.38
	Southern part	181 (24.4%)	
	Central part	136 (18.3%)	
	Eastern part	146 (19.7%)	
	Western part	138 (18.6%)	

Table 2 provides insights into dental professionals’ practices and preferences regarding implant procedures. The majority (584, 78.7%) of the 742 participants reported involvement in implant placement, emphasizing its significance in modern dental practice. Similarly, a substantial proportion (541, 72.9%) of individuals were engaged in implant restoration, highlighting its crucial role in ensuring implant-supported prosthesis functionality. Conversely, the remaining 125 (21.4%) respondents indicated non-engagement in restoration procedures, suggesting varied practice patterns or specializations. Regarding implant treatment planning, a high level of engagement was observed, with 459 (78.6%) individuals actively participating. This underscores the collaborative nature of treatment planning in achieving optimal patient outcomes. Preferences for abutments varied, reflecting individual preferences and clinical considerations. Choices ranged from pre-fabricated metal and ceramic abutments to angulated, cast-to-gold/UCLA, and computer-aided design/computer-aided manufacturing abutments. Similarly, preferences for abutments in implant-supported fixed dental prostheses and implant-supported/retained dentures varied based on clinical requirements and patient preferences. In both single implant cases and implant-supported fixed dental prosthesis cases, screw-retained restorations were favored by a majority of participants. This preference is attributed to perceived advantages such as retrievability, ease of maintenance, and potential for minimizing complications associated with cement-retained restorations.

**Table 2:** Variables pertaining to implant types analyzed. CAD/CAM = computer-aided design/computer-aided manufacturing

Variable type analyzed	Frequency n (%)	Chi-square value
Were implants placed by participants?	Yes: 584 (78.7%)	25.67
	No: 158 (21.3%)	
Were implants restored by participants?	Yes: 541 (72.9%)	20.54
	No: 201 (27.1%)	
Participation in implant treatment planning	Yes: 459 (78.6%)	18.92
	No: 125 (21.4%)	
Usage of implant planning software	Always: 174 (29.8%)	15.78
	Use only for special cases: 242 (41.4%)	
	Limited use/do not use at all: 168 (28.8%)	
Type of abutments for single implant-supported crowns	Pre-fabricated metal (Ti, gold): 120 (20.5%)	13.25
	Pre-fabricated ceramic (zirconia, alumina): 112 (19.2%)	
	Angulated abutments: 118 (20.2%)	
	Cast-to-gold/UCLA abutments: 134 (23.0%)	
	CAD/CAM abutments: 100 (17.1%)	
Type of abutments for implant-supported fixed dental prosthesis	Pre-fabricated metal (Ti, gold): 110 (18.9%)	10.76
	Pre-fabricated ceramic (zirconia, alumina): 108 (18.5%)	
	Angulated abutments: 116 (19.9%)	
	Cast-to-gold/UCLA abutments: 130 (22.3%)	
	CAD/CAM abutments: 120 (20.5%)	
Type of attachments for implant-supported/retained denture	Bar and clip attachment: 124 (22.9%)	11.87
	Ball and socket attachment: 116 (21.4%)	
	Locator attachment: 110 (20.3%)	
	Telescopic attachment: 101 (18.6%)	
	Magnetic attachment: 90 (16.6%)	
Type of fixed implant restorations in single implant cases	Screw-retained: 309 (57.1%)	30.12
	Cement-retained: 232 (42.9%)	
Type of fixed implant restorations in implant-supported fixed dental prosthesis cases	Screw-retained: 295 (54.5%)	28.67
	Cement-retained: 246 (45.5%)	

The preferred loading protocols for different clinical conditions are shown in Table 3. For anterior teeth (incisors and canines), early implant loading was favored by 260 participants, likely due to the desire to expedite treatment timelines while ensuring favorable esthetic outcomes. Immediate loading, chosen by 146 participants, is gaining acceptance for rapid restoration and functional rehabilitation in the aesthetic zone. Conventional loading, preferred by 178 participants, remains relevant, especially when stringent criteria for implant stability and osseointegration are emphasized. In contrast, for posterior teeth (premolars and molars), immediate loading was preferred by 235 participants, highlighting its benefits in optimizing patient comfort and function, particularly in load-bearing areas. Early implant loading, chosen by 200 participants, and conventional loading, preferred by 149 participants, offer alternative approaches based on clinical considerations and patient needs. In the case of edentulous patients, immediate loading was the most favored option, with 183 participants expressing a preference for this protocol. This reflects the growing acceptance of immediate loading protocols in full-arch rehabilitation, providing expedited restoration and enhanced patient satisfaction. Early implant loading, preferred by 222 participants, and conventional loading, chosen by 179 participants, remain viable options, especially in cases requiring staged treatment protocols.

**Table 3:** Preferred loading protocol (where n = number of samples).

Condition	Immediate loading (n)	Early implant loading (n)	Conventional loading (n)	Chi-square value	F-value
Anterior (incisors and canines)	146	260	178	12.37	7.89
Posterior (premolars and molars)	235	200	149	9.84	6.21
Edentulous patients	183	222	179	8.96	5.67

**Figure 1:**
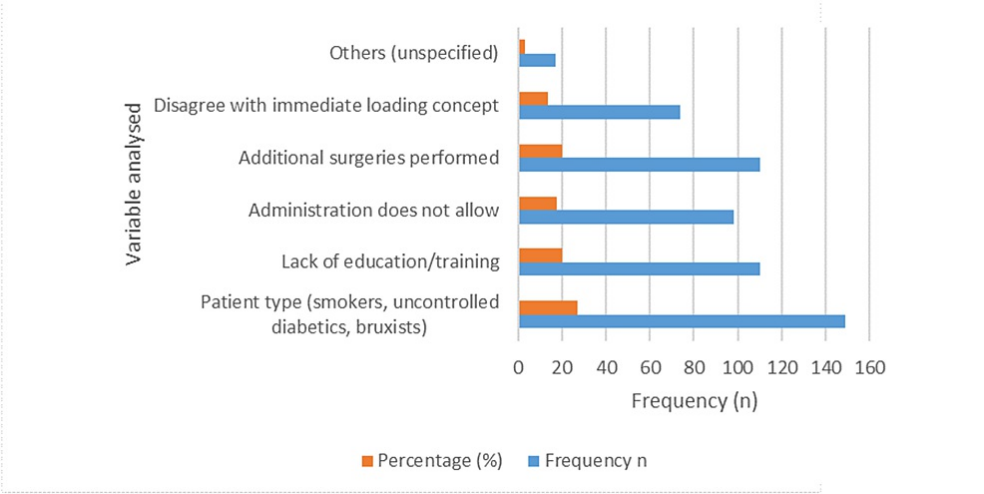
Main reasons for not using immediate loading.

Figure 1 offers insights into the factors influencing clinical decision-making among the respondents. Patient-specific factors, including smoking habits, uncontrolled diabetes, and bruxism, were cited by 149 (27.0%) participants, emphasizing the importance of individual patient assessments and risk management in treatment planning to ensure long-term implant success. Concerns regarding the lack of education or training were expressed by 110 (19.9%) participants, indicating a need for ongoing professional development to enhance proficiency and confidence, particularly in adopting advanced treatment protocols such as immediate loading. Similarly, the perceived need for additional surgeries was cited by an equal number of participants, highlighting the complexity of implant therapy and the importance of comprehensive preoperative evaluations. Institutional factors, such as administrative policies prohibiting immediate loading, were mentioned by 98 (17.8%) participants, underscoring the impact of organizational protocols on clinical decision-making. Some individuals expressed skepticism or disagreement with the concept of immediate loading, with around 56 (13.4%) respondents reflecting ongoing debate and variability in clinical opinions. This highlights the necessity for further research and evidence-based guidelines to inform clinical practice. Additionally, 7 (3.1%) participants cited unspecified reasons for abstaining from immediate loading, indicating the intricate nature of clinical decision-making and the diverse array of variables influencing treatment planning.

In Table 4, the preferred implant systems for different dental conditions are outlined, representing the diverse choices made by respondents of this study. For anterior teeth (incisors and canines), Straumann implants were most favored, utilized by 75 participants. This preference likely reflects Straumann’s reputation for achieving esthetic outcomes in the anterior region. Ankylos and Nobel Biocare implants followed, each utilized by 70 participants, indicating a competitive landscape in implant dentistry with multiple viable options. Mini implants were the least preferred for anterior conditions, utilized by 45 participants, possibly due to concerns about long-term stability. For posterior teeth (premolars and molars), Astra Tech implants were preferred, utilized by 75 participants, likely due to their strong osseointegration and long-term outcomes in regions subject to higher occlusal forces. Bio Horizon and Ankylos implants followed closely, chosen by 70 and 68 participants, respectively. Once again, mini implants were the least favored for posterior conditions, utilized by 55 participants. In edentulous patients, Ankylos, Nobel Biocare, and Straumann implants were most commonly utilized, each preferred by 70 participants, reflecting their versatility and reliability in full-arch restorations. Mini implants were the least favored, utilized by 55 participants, suggesting caution in their use due to concerns about stability. Straumann emerged as the most favored implant system, chosen by 75 participants, followed closely by Ankylos, chosen by 72 participants. Mini implants were the least favored overall, chosen by 55 participants, indicating a prevailing cautious approach despite their potential benefits in certain scenarios.

**Table 4:** Preferred implant system analyzed (where n = number of samples).

Condition	Astra Tech (n)	Ankylos (n)	Bio Horizon (n)	Neoss (n)	Biomet 3i (n)	Nobel Biocare (n)	Mini Implant (n)	Straumann (n)	Zimmer (n)	Other (n)	Chi-square value	F-value
Anterior (incisor and canines)	65	70	60	55	58	67	45	75	50	55	8.34	5.67
Posteriors (premolars and molars)	75	68	70	58	60	65	55	72	52	55	9.21	6.78
Edentulous patients	68	70	65	58	60	65	55	70	52	57	7.89	5.34
Overall preference (first choice)	70	72	65	60	62	70	55	75	58	63	10.45	7.21

The assessment of statistical variables pertaining to implant types has been outlined in Table 5. Significant associations were observed between the placement of implants and key variables such as whether implants were restored, participation in implant treatment planning, and the type of abutments used. These findings underscore the interconnectedness of different stages in the implant therapy process, emphasizing the importance of comprehensive treatment planning and interdisciplinary collaboration for optimal patient outcomes. Additionally, the use of implant planning software was significantly associated with implant placement, highlighting the role of technological advancements in improving precision and efficiency in implant procedures. This underscores the increasing reliance on digital tools in modern implant dentistry, leading to more predictable outcomes and streamlined workflows. Furthermore, significant associations were found between the type of attachments used for implant-supported dentures and the placement of implants, as well as between the type of fixed implant restorations and implant placement in both single implant cases and implant-supported fixed dental prosthesis cases. These associations emphasize the need to tailor treatment approaches to individual patient needs and clinical considerations, reflecting the nuanced decision-making process involved in selecting appropriate implant systems and prosthetic components.

**Table 5:** Variables pertaining to the implant types analyzed.

Variable pair	P-value
Implants placed vs. restored	0.0002
Implants placed vs. planning	0.0001
Implants placed vs. software	0.042
Implants placed vs. abutments	0.0003
Implants placed vs. attachments	0.009
Implants placed vs. restorations (single)	0.0004
Implants placed vs. restorations (fixed)	0.0006

**Figure 2:**
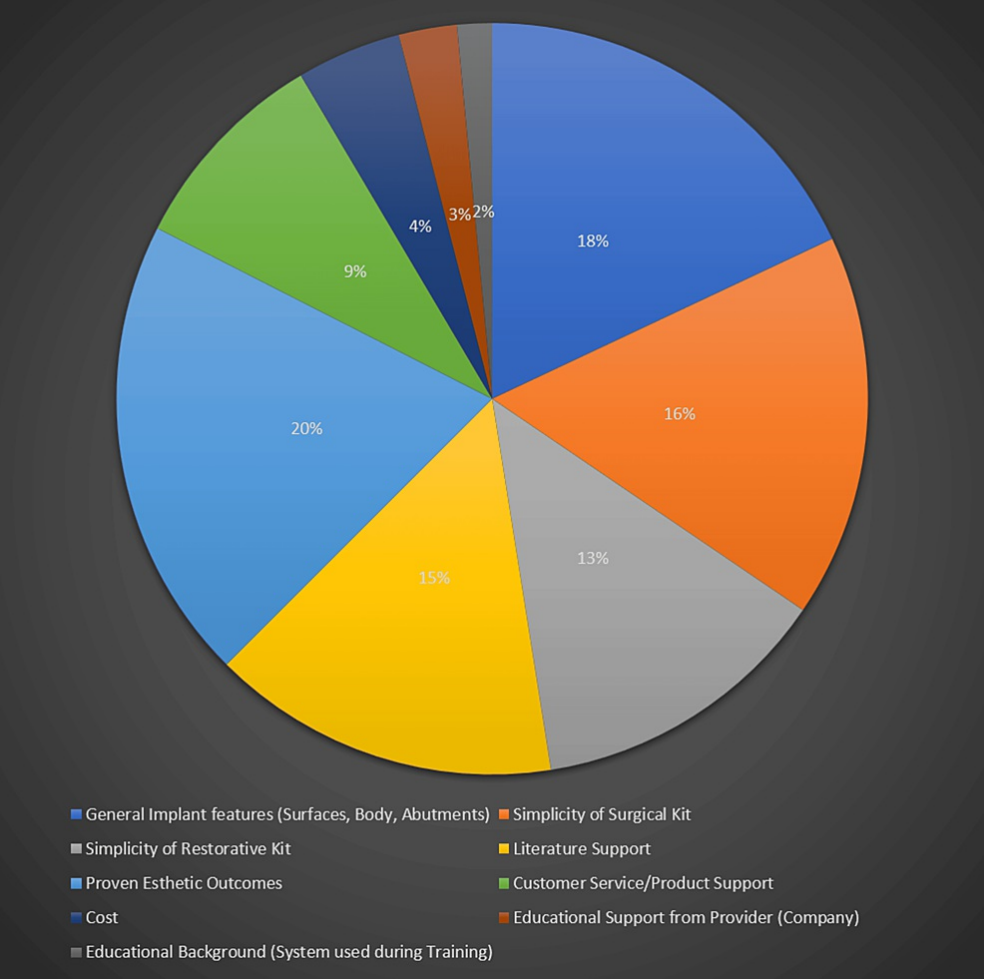
Most important factors when selecting an implant system (in percentage).

Figure 2 depicts insights into the key criteria influencing the selection of implant systems among participants, shedding light on the diverse factors shaping clinical decision-making among the assessed sample size in this study. Emphasis on proven aesthetic outcomes was noted as the most important criterion by 148 (20%) participants, reflecting the high priority placed on achieving natural-looking dental restorations in implant therapy. General implant features, including surfaces, body design, and abutments, were considered significant by 133 (18%) participants, highlighting the importance of technical specifications and design characteristics in guiding implant system selection. The simplicity of the surgical kit ranked third in importance, chosen by 122 (16.5%) participants, underscoring the practical benefits of streamlined surgical workflows and instrumentation in facilitating efficient and predictable treatment delivery. Literature support was identified as the fourth most important criterion by 111 (15%) participants, emphasizing the role of evidence-based practice and clinical research in informing treatment decisions and ensuring optimal patient outcomes. Educational background, including the system used during training, was perceived as the least important criterion, chosen by only 11 (1.5%) participants, suggesting its limited influence on ongoing clinical practice and decision-making processes. Similarly, educational support from the provider or company ranked eighth in importance, chosen by 2.5% of participants, while cost considerations ranked seventh, chosen by 33 (4.5%) participants, indicating the complex balance between clinical efficacy, practical considerations, and financial constraints in implant system selection.

## Discussion

Most respondents in our study had experience placing (78.7%) and restoring (72.9%) implants. We also assessed the use of implant planning software, with a minority (29.8%) always using it, while the rest used it for special cases (41.4%) or made little or no use of it (28.8%). Preferences for implant loading protocols were also examined, with early implant loading being the most common for anterior teeth and immediate loading being most popular for posterior teeth and edentulous patients. The main reason cited for not using immediate loading was patient-specific factors such as smoking or diabetes. When choosing an implant system, the most important factor in our investigation was deemed to be proven aesthetic outcomes, followed by general implant features and the simplicity of the surgical kit. The least important factors were the system used during training and educational support from the provider or company. Cost was the seventh-most important criterion.

Our study and those of Mously et al. [14] and Al Saleh et al. [1] provide similar insights into dental implant practices, though with distinct focuses and findings. Mously et al.’s study [14] found that the level of knowledge about tooth replacement methods was relatively low (56%), with only 44.4% having heard about implants, bridges, and dentures. In contrast, our study revealed high levels of engagement with implant placement and restoration among surveyed dentists. The difference underscores the disparity in knowledge and experience between the general public and dental professionals. Both studies, however, highlighted the importance of education, with Mously et al. [14] noting that knowledge about dental implants was the highest among those with postgraduate education. Al Saleh et al.’s study [1], on the other hand, aligned more closely with our research, focusing on prosthodontic specialists in Dubai. A high percentage (84.6%) of the registered prosthodontists completed their survey, with 66.2% practicing implant dentistry, which is lower than our findings of 78.7% placing and 72.9% restoring implants. Interestingly, their study found a preference for prefabricated metal abutments and screw retention, specific elements not covered in our survey. Unlike our study, which indicated variable preferences for loading protocols depending on tooth position, Al Saleh et al. [1] reported that conventional loading was the most selected type of loading in all oral conditions. This difference may reflect regional variations in practice or differences in patient demographics. Alqahtani et al.’s [13] study focused on older patients (>58 years) who had not undergone any implant or bone graft procedures. This focus contrasts with our study, which surveyed dental practitioners rather than patients. Alqahtani et al. found that knowledge related to dental implants was low among their population, with only 4.5% showing good knowledge. This aligns with our finding that less than a third of practitioners consistently used implant planning software, suggesting a gap in both patient and practitioner knowledge. A significant number (49.6%) of Alqahtani et al.’s [13] participants were willing to replace their current prosthesis with dental implants, indicating an openness to this procedure despite the low knowledge levels. Saad et al.'s study [15], on the other hand, surveyed undergraduate students, interns, and freshly graduated dentists in Saudi Arabia. Their findings showed a better level of knowledge regarding dental implant procedures (37.5%) and implant complications (38.9%) compared to Alqahtani et al.’s patient population. This likely reflects the advanced education and training of the surveyed group in Saad et al.’s study [15]. Most participants in Saad et al.’s study recognized case selection (54.17%) as the most important cause of complications associated with dental implants, highlighting the importance of patient-specific treatment planning, a theme also emphasized in our study.

Dental implants have become a universally recognized solution for the prosthetic treatment of fully or partially toothless patients. They are particularly advantageous for older patients when implemented with careful diagnosis and appropriate procedure planning, serving as an effective method for tooth replacement [16]. However, existing research indicates that the usage rate of dental implants within the elderly demographic is lower than that of other age groups [17,18]. International data suggest that the majority of dental implant placements occur within the age range of 60 to 75 years, demonstrating the procedure’s acceptance within this demographic [19-22]. The prevalence of dental implant usage within the senior population of Saudi Arabia is currently imprecise due to a lack of sufficient data [23]. Despite the increasing popularity of dental implants as a treatment option for toothlessness among the Saudi population, owing to the associated benefits of improved denture stability, enhanced functionality, and improved quality of life, recent studies within the region have not sufficiently included geriatric dental patients [24,25]. Additionally, regional studies have identified a gap in awareness regarding the advantages of such treatments. The necessity of implants becomes even more pronounced in this region when you factor in the fact that the incidence of tooth loss within the adult population of Saudi Arabia is notably high [26-28]. Research indicates that 69% of this demographic have at least one missing tooth, while approximately 2.6% are fully edentulous [26-28].

The limitations of this study are primarily related to the self-reported nature of the data, the geographical reach, and the scope of the variables studied. The study relied on self-reported data for its findings, which inherently brings about a potential for recall bias and subjective interpretations. Participants’ recall of their practices, preferences, and reasons for certain choices might not have been entirely accurate, which could have influenced the results. The geographical reach of the study, focusing on Saudi Arabian dentists, is another limitation. The findings, therefore, may not be universally applicable or generalizable to other populations of dentists worldwide due to variations in educational training, practice settings, and cultural or societal influences on dental care. Further, while the study did analyze a variety of variables related to implant procedures, its scope was still somewhat limited. For instance, the study did not investigate all potential factors that could influence a dentist’s choice of an implant system or loading protocol, such as patient demographics, socioeconomic factors, or specific clinical scenarios. Additionally, the responses related to the non-usage of immediate loading might have been influenced by multiple factors that were not considered in this study. For instance, the reasons for not using immediate loading could have been affected by organizational policies, personal beliefs, or a lack of resources. The study did not delve deeper into these possibilities, which could have provided a more nuanced understanding of the reasons behind the choices made by the participants.

## Conclusions

Most practitioners had placed and restored implants, as per the findings, which offer important insights into dental implant practices. Although many used implant planning software for intricate procedures, there was a suggestion that it could be used more widely. The loading techniques used were sophisticated and tailored to individual dental problems. For front teeth, early implant loading was preferred; for posterior teeth and edentulous individuals, rapid loading was recommended. Patient type, lack of training, extra operations, and administrative limitations were among the obstacles to adopting quick loading. The dental condition affected the choice of implant systems. Practitioners preferred Straumann implants for anterior teeth and Astra Tech for posterior teeth. Ankylos, Nobel Biocare, and Straumann implants were equally preferred for edentulous patients. All things considered, the Straumann implant system was the most popular. Overall, this study emphasizes how important it is to prioritize practical outcomes and patient happiness when making decisions. Thus, the results provide a thorough understanding of dental implant procedures, highlighting the significance of dental problems, patient characteristics, and cosmetic results in these procedures.
